# Assessment of the Diagnostic Methods of Mizaj in Persian Medicine: A Systematic Review

**DOI:** 10.3390/diagnostics13050818

**Published:** 2023-02-21

**Authors:** Mostafa Ahmadi, Hoda Shirafkan, Seyyed Ali Mozaffarpur

**Affiliations:** 1 Student Research Committee, Babol University of Medical Sciences, Babol, Iran; 2 Social Determinants of Health Research Center, Health Research Institute, Babol University of Medical Sciences, Babol, Iran; 3 Traditional Medicine and History of Medical Sciences Research Center, Health Research Institute, Babol University of Medical Sciences, Babol, Iran

**Keywords:** Persian medicine, temperament, traditional medicine, questionnaire, diagnosis

## Abstract

The concept of mizaj corresponds to the idea of personalized medicine and is the main diagnostic principle of Persian medicine (PM). This study aims to investigate diagnostic tools for the detection of mizaj in PM. In this systematic review of articles published before September 2022, articles were searched for in the Web of Science, PubMed, Scopus, Google Scholar, SID databases, and gray literature. The titles of the articles were screened by researchers and relevant articles were selected. Abstracts were reviewed by two reviewers to select final articles. Subsequently, the articles found were critically evaluated by two reviewers according to the CEBM method. Finally, article data were extracted. Of the 1812 articles found, 54 were included in the final evaluation. Of these, 47 articles were related to the diagnosis of whole body mizaj (WBM). WBM was diagnosed in 37 studies using questionnaires and 10 using expert panels. In addition, six articles examined the mizaj of organs. Only four of these questionnaires were found with reported reliability and validity. There were two questionnaires for assessing WBM, but neither had sufficient reliability and validity. Questionnaires that assess organs had weak designs and lacked sufficient reliability and validity.

## 1. Introduction

Traditional and complementary medicine paradigms are used increasingly worldwide. In recent years, many studies in the field of complementary medicine have been published in Medline [1,2]. In addition, there has recently been a trend toward person-centered medicine [3]. Personalized medicine means that people are evaluated based on individual characteristics that can influence disease manifestations and treatment patterns [4]. Although this paradigm is a new approach in current medicine, it has been the basis of diagnosis and treatment in traditional Chinese medicine, Ayurveda, and Persian medicine (PM) for centuries [5,6].

PM is one of the traditional schools of medicine where the diagnosis and treatment are based on the concept of mizaj (also known as temperament). Mizaj is determined based on the physical, physiological, and psychological characteristics of individuals [7]. Based on this concept, according to 10 criteria, each person belongs to one of four simple groups (hot, cold, dry, wet), four complexes (cold and wet, hot and wet, cold and dry, hot and dry), or moderate mizaj [8,9,10].

From the point of view of PM, each person and also each organ of the body has its own mizaj. Consequently, the resultant mizaj of the organs (including the brain, heart, and liver) makes the whole body mizaj (WBM) [11]. It is believed that when the WBM and the mizaj of all the organs are in balance, there is health in the individual’s body. Any imbalance in WBM or the mizaj of organs can lead to illness (called *sue-mizaj* or dystemperament in PM) [10]. PM references provide qualitative and descriptive criteria for determining the WBM and its main organs [9].

Several studies have recently used different methods to identify mizaj. Some studies used expert panels, while others used questionnaires as the basis for diagnosis. Some studies have investigated the relationship between some diseases and WBM and organ mizaj. These studies evaluated the correlations of mizaj with some objective indicators [12,13,14,15]. However, it seems that different invalid and unreliable methods were used in these studies. Based on our searches, no study has been conducted to investigate the diagnostic tools in PM. This study aims to investigate the diagnostic tools for determining mizaj in PM.

## 2. Materials and Methods

This systematic review was conducted in 2022 in Babol, Iran. Articles in Persian and English were selected for review.

### 2.1. Information Sources

English electronic databases including the Web of Science, PubMed, Scopus, Google Scholar (the first 20 pages), and SID databases (in Persian) and gray literature up to 2022 September were searched.

### 2.2. Eligibility Criteria

Our review includes all types of studies (cross-section, case–control, cohort, RCT) using standard tools for mizaj assessment, mizaj expert opinion, and human studies on mizaj evaluation for all participants in any age group, sex, race, etc. Animals, paraclinical articles, and low-quality articles based on critical appraisal tools were excluded from our study.

### 2.3. Search Strategy

In the absence of a specific word suitable for the concept of mizaj in MeSH terms, the words used in the title and keywords were extracted in the initial search. Then, the final words were selected for the search based on the opinion of PM experts. A search strategy was developed based on the keywords found, and the search was conducted by abstract and title. The databases query syntaxes are shown in the Supplementary Data.

### 2.4. Selection and Data Collection Process

Researchers screened the titles of the found articles, and selected related articles to review their summary. Articles’ abstracts were reviewed by two reviewers and the final articles were selected. References of final articles were also searched for relevant articles or gray literature. Articles on Unani medicine not related to PM, systematic reviews, animal studies, phytotherapy articles without mizaj diagnosis, PM studies using methods other than mizaj diagnosis, book chapters, letters, and case report articles were excluded from the study.

### 2.5. Risk of Bias Assessment

Then, the found articles were critically reviewed by two reviewers using the Oxford Centre for Evidence-Based Medicine critical appraisal tools (CEBM) [16]. If the two reviewers disagreed, a consensus was reached in the presence of a third reviewer. The articles that were not of sufficient quality to be included in the study were excluded, with the agreement of the reviewers.

### 2.6. Data Items

Data including author names, date and location of study, year of publication, type of the study, sample size, age range or mean age, type of mizaj (WBM or organ mizaj), mizaj assessment tools’ reliability and reliability values, and the number of questions of the tool were extracted from the articles.

### 2.7. Data Synthesis

Data synthesis was performed by examining the text in the results section line by line, discussing it, and then identifying the proper items. Finally, data from the articles were extracted and summarized in the tables.

## 3. Results

### 3.1. Characteristics of the Included Studies

**Figure 1:**
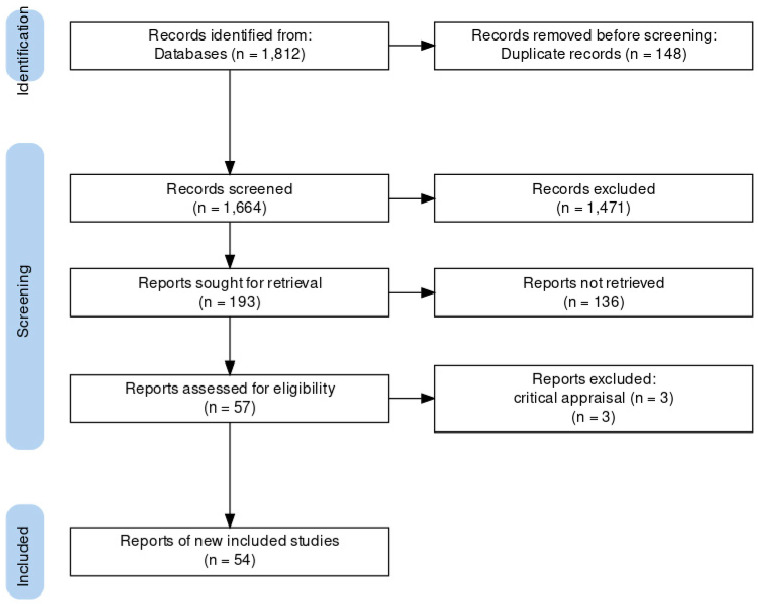
PRISMA 2020 flow diagram of included studies.

In this study, by searching the electronic databases, 1812 articles were found, out of which 148 articles were removed due to similarity. After excluding the articles according to the exclusion criteria, 57 studies remained. (Figure 1).

**Figure 2:**
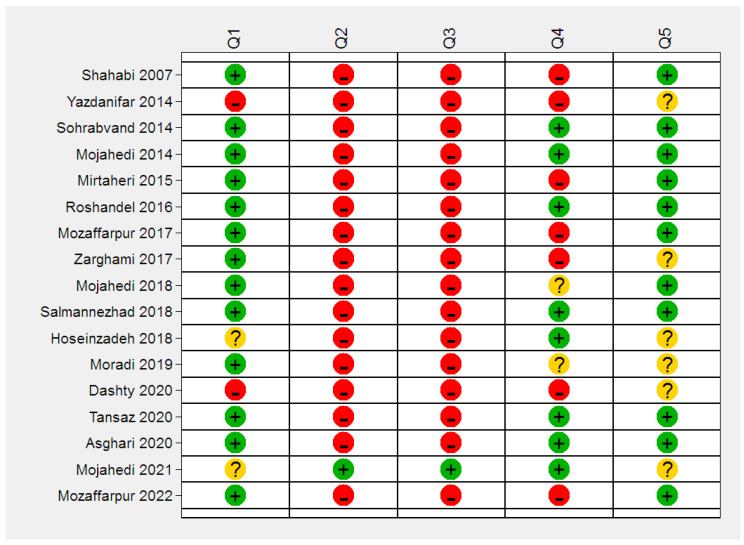
Critical appraisal of primarily included studies. Q1 = Was the diagnostic test evaluated in a representative spectrum of patients (like those on whom it would be used in practice)? Q2 = Was the reference standard applied regardless of the index test result? Q3 = Was there an independent blind comparison between the index test and an appropriate reference (‘gold’) standard of diagnosis? Q4 = Are test characteristics presented? Q5 = Were the methods for performing the test described in sufficient detail to permit replication? Each row of the figure corresponds to a critical evaluation of the articles: Shahabi [8], Yazdanifar [18], Sohrabvand [25], Mojahedi [5], Mirtaheri [26], Roshandel [29], Mozaffarpur [36], Zarghami [17], Mojahedi [41], Salmannezhad [20], Hoseinzadeh [23], Moradi [44], Dashty [19], Tansaz [22], Asghari [53], Mojahedi [60], Mozaffarpur [67]. 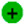 = Yes, 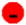 = NO, 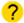 = Unclear.

**Figure 3:**
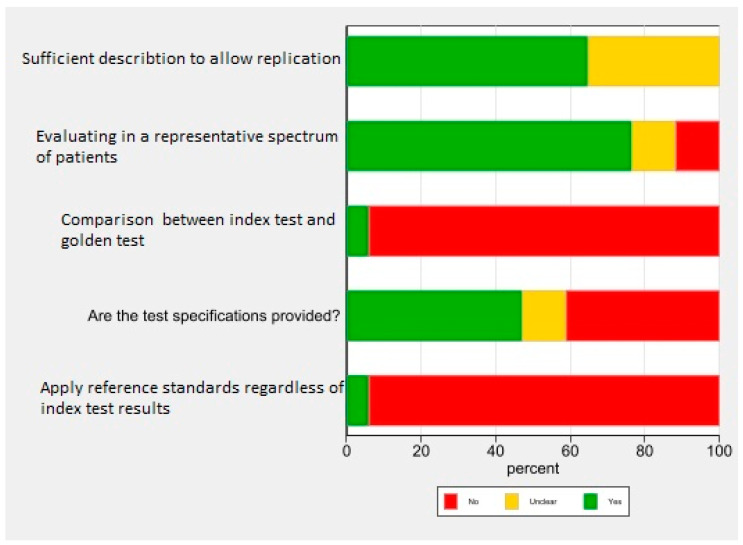
Critical appraisal chart.

Then, the articles were subjected to critical appraisal to evaluate their quality (Figure 2 and Figure 3). According to the consensus of two reviewers, three articles by Dashty, Yazdanifaro, and Zarghami were excluded from the review. Dashti and Yazdanifar’s articles did not receive a score for the most important feature, which is related to the random selection of the sample and the appropriate selection of the sample, and also did not provide a proper explanation of the characteristics of the test. The method of performing the test in patients is not fully explained. Zarghami’s article did not provide specifications that determine the sensitivity and specificity of the test. On the other hand, the test was not performed in a suitable range of society and the sampling method was not random [17,18,19].

Thus, 54 articles were included in the final review of the study.

As most of the studies used some limited questionnaires (Mojahedi, Salmannejad, etc.), critical appraisal using the CEBM tool was performed for articles using the new method of mizaj assessment.

Due to the lack of a standard mizaj questionnaire, most of the second and third columns after the assessment are red.

### 3.2. Mizaj Determination

Out of the 54 articles included in the study, 47 articles were related to the diagnosis of WBM. The diagnosis of WBM in 37 studies was made using a questionnaire, of which 30 studies used the Mojahedi questionnaire [5] and 7 used the Salmannejad questionnaire [20]. In addition, in 10 studies, an expert panel was used to determine the mizaj. Meanwhile, there is a study that used both the Mojahedi questionnaire and an expert panel to assess WBM [21].

Out of the 54 articles found, six articles investigated the organ mizaj. Four articles involved uterine mizaj, one study used a scientifically developed questionnaire to assess the uterine mizaj [22], and the other two studies used a researcher-developed questionnaire to assess uterine mizaj.

One of the studies to assess organ mizaj was related to the mizaj of the brain and another to the dystemperament of the digestive system [12,23]. Except for one study conducted in India using the Mojahedi questionnaire [24], all other studies were conducted in Iran (Table 1).

After reviewing the articles found, only four questionnaires were found with reported reliability and validity (Table 2).

### 3.3. Mizaj Assessment Tools

Novel questionnaires were introduced in four studies and were used in other studies. They include:

#### 3.3.1. Mojahedi Questionnaire [5]

Developed by a group of 10 PM experts, it is a self-report questionnaire containing 10 items (8 in hotness/coldness and 2 in dryness/wetness). It was designed in 2012 to 2013 in Tehran, Iran, to assess WBM in 20–40 healthy volunteers. The researchers had a good design to develop the scale and assessed the reliability and validity of the questionnaire. It is the most widely used WBM assessment tool in mizaj studies (28 out of 49 studies including review studies). Its internal consistency is 0.7, but the average consistency of the fields of the questionnaire is 55.5% (hotness: 65%, coldness: 52%, dryness: 53%, wetness: 53%).

#### 3.3.2. Salmannejad Questionnaire [20]

A well-designed self-report questionnaire with 20 items (15 in hotness/coldness and 5 in dryness/wetness) was developed by a group of 15 PM experts in 2015–2017 in Babol, northern Iran. It can assess WBM in 20–60-year-old individuals. It was used in 4 out of the 49 included studies in this review. More items of the 10 criteria of the mizaj assessment (based on PM references) were used in this study.

#### 3.3.3. Hoseinzadeh Questionnaire [23]

It is a quantitative tool for the diagnosis of gastrointestinal dystemperaments. The items were generated through an expert panel and literature review, and then given weight after the item reduction process. The researchers then designed software to calculate them. Its reliability is stated to be evaluated, but only internal consistency (standardized Cronbach’s alpha) is reported. This means that the usual method of assessing reliability (test–retest) was not performed. The interpretation of the total score is also not explained. Furthermore, no cut-off point for the questionnaire is calculated, and the sensitivity and specificity of this tool are not reported. More importantly, the items of hotness/coldness and dryness/wetness of mizaj of gastrointestinal dystemperaments are not addressed in this study.

#### 3.3.4. Tansaz Questionnaire [22]

It is a 12-item questionnaire to evaluate the uterine temperaments (mizaj) of infertile women in Iran. Nine of the items assess the hotness/coldness of the uterine mizaj and three of them assess the dryness/wetness. Item generation was performed using PM references. The rest of the method seems logical. Acceptable internal consistency, reliability, and validity are reported. However, the process of calculating cut-off points is ambiguous when interpreting the scores. Furthermore, no sensitivity and specificity as important indices are reported.

### 3.4. Expert Panel Method in Mizaj Assessment

Some studies evaluated the mizaj using expert panels. Most of them made criteria based on PM references and then 1 to 15 experts evaluated the mizaj. Novel methods of expert panels were used in only two studies:

#### 3.4.1. Asghari Method [53]

In this study, three PM experts (MD. Ph.D.) with at least 5 years of clinical practice experience with a break of at least 2 weeks visited 30 volunteers in two different sessions. Mizaj assessments were performed separately by experts for each participant and recorded on a sheet; then to finalize the diagnosis, an expert panel discussion was held.

#### 3.4.2. Mizaj Assessment Methods in Amirkola Health and Aging Project (AHAP Cohort) [11]

This process was carried out on 2135 elderly people in two phases. In the first phase, 5 to 10 elderly people were examined daily by one PM expert for 20 min, and videos and audio files were recorded. At this phase, a researcher-made 74-question checklist was fulfilled and then the WBM and main organ mizaj were determined. Finally, the elderly people with a typical diagnosis of WBM (based on PM professional sentiment and expertise) were noted. The diagnosis was determined based on the clinical experience of the PM experts. At the end of this phase, 268 elderly people were identified as typical. Their files were evaluated in the second phase (expert panel sessions). In the second phase, the files of the elderly people with typical diagnoses were evaluated in an expert panel with the presence of five PM experts for an average of 30 to 45 min. At first, the expert who visited the elderly person introduced the person without revealing the diagnosis. The audio and video files recorded by the examiner were then played on a TV set. Finally, all five present experts, without any discussion, recorded their diagnoses secretly and individually. Complete agreement was considered if at least four of the five experts made the same diagnosis (206 people). Otherwise, relative agreement or disagreement was considered.

## 4. Discussion

According to the research strategy, a total of 1812 articles were found in the search in the electronic databases. After the process of the systematic review, 54 articles were finally included in the study.

Among 45 articles in the field of WBM, 37 articles were conducted using the Mojahedi or Salmannejad mizaj assessment tools, which reported reliability and validity. The Mojahedi questionnaire [5] is widely used in WBM assessment studies. Its internal consistency is acceptable (0.7), but the average consistency of the fields of the questionnaire is 55.5% (hotness: 65%, coldness: 52%, dryness: 53%, wetness: 53%). As an explanation, if the sensitivity is 50%, there are as many true positives as false negatives, indicating that the test is not useful in determining the true diagnosis [69]. Additionally, as the minimum acceptable sensitivity + specificity value is 1.5 [70], it can only reach the minimum acceptable score in hotness, and cannot make a true diagnosis in coldness, dryness, and wetness. This may be because the final questionnaire does not use some indices that are important for mizaj assessment based on PM references. Therefore, the experts used these indices (as the gold standard in this study) to assess mizaj, but they were not used in the questionnaire. In this questionnaire, the moderate is so narrow that most of the population will be categorized as hot or cold mizaj or as dry or wet mizaj, which is not matched with the real diagnosis based on experts and references. Additionally, in dryness/wetness, only two items out of 10 indices of PM references are used and the other eight types of indices are dismissed. The sensitivities of moderate mizaj have not been reported, neither in hotness/coldness nor in dryness/wetness. This questionnaire is developed to identify the mizaj of healthy individuals. However, it has been used in some studies at younger or older ages or in diagnosing the mizaj of unhealthy people [12,24,43,54,61,66]. This means that in these studies, it was used in areas for which it was not developed.

Compared with the Mojahedi questionnaire, the Salmannejad questionnaire [20] has a wider age range (20–60 years old). In this study, sensitivities of moderates in hotness/coldness and dryness/wetness were evaluated. Since this questionnaire has more items than the Mojahedi questionnaire, the ranges of moderates are wider. Although the questionnaire has acceptable internal consistency, in subgroups other than dryness, which has an acceptable minimum sensitivity + sensitivity score (>1.5), other factors (hotness, coldness, wetness, and moderates) did not reach the minimum expectable values. Compared with the Mojahedi questionnaire, the Salmannejad questionnaire has higher sensitivity and lower specificity generally. This means that the Salmannejad questionnaire is better for screening mizaj in research.

Out of six documents found on the diagnosis of organ mizaj, one article was on the diagnosis of digestive mizaj, four articles were on the diagnosis of uterine mizaj, and one article was on the diagnosis of brain mizaj. None of these studies took scientific steps to develop valid and reliable questionnaires. In most of them, items were generated and used to determine mizaj based on the opinion of the researchers and using the PM references. Two of them introduced a questionnaire. The Hoseinzaheh questionnaire that was developed for the diagnosis of gastrointestinal dystemperaments is interpreted as weak because, except for internal consistency, the usual methods of assessing reliability (test–retest) and validity are not reported. Additionally, in Tansaz’s questionnaire on uterine mizaj, its sensitivity and specificity are not reported. Therefore, this questionnaire is also interpreted as weak.

In studies that assessed mizaj based on an expert panel model, only the Asghari method [53] and AHAP method [11] introduced a clear method. None of these studies can provide a standard model for mizaj assessment.

Our review found some other types of studies that are related to the concept of the mizaj but did not meet our inclusion criteria. Most of them are preliminary studies to develop new valid and reliable diagnostic tools. The first category is articles that help conceptualizations. As the goal of our study was the practical method of evaluating mizaj, these articles were removed during the study [7,71,72,73,74]. The second type of these studies are the articles that evaluated the current situation of mizaj assessment, in the absence of valid and reliable diagnostic tools [36]. These articles can help to monitor the validity and reliability of the mizaj assessment over time. The third type of the studies aimed to give weight to each proposed index of mizaj assessment (based on PM references) to develop a final diagnostic tool. Some of these studies evaluated the unconscious effect of indices on the mizaj assessment [41]. In some other studies, PM experts were asked to give weight to each index (10 criteria of mizaj assessment based on PM references) [75]. In some other studies of this type, each proposed criterion was evaluated in correlation with the final diagnosis of mizaj [34].

Our study had some limitations. Although all the articles that used the PM-based mizaj assessment methods were reviewed, the details of the methods of mizaj assessment were not given in most of the articles. The limitation of language was another limitation of our search strategy. Based on our prediction, the entire articles should be published in Persian or English, but it may be helpful to be able to search in other languages. The lack of definite words related to the concept of mizaj in the MeSH database was another limitation of our study. Therefore, in the preliminary primary search stage, we tried to extract related keywords from related articles.

Based on our systematic review, there is still no valid and reliable tool or questionnaire as the gold standard in PM. Designing and developing new diagnostic tools to identify mizaj in both WBM and organs is strongly suggested. We also propose that future studies use new statistical methods that are used in the field of personalized medicine to assess the relationship between paraclinical criteria and mizaj.

We also propose assessing all physiological parameters in healthy individuals and defining the companionship or interrelationships of these parameters. This pathway can be used as a parallel model to establish a gold standard for categorizing people. This means that all clinical and paraclinical parameters can be used in this way, rather than limiting ourselves to PM reference criteria for determining the mizaj. Additionally, the mizaj assessment may require evaluation of the correlation between a set of indicators and the final diagnosis, and it is not sufficient to check each criterion individually with the final diagnosis.

## 5. Conclusions

In this systematic review, we found two questionnaires for evaluating WBM, neither of which is sufficiently reliable and valid. Two other questionnaires for organ mizaj assessment are poorly designed and lack sufficient reliability and validity. Developing a new valid tool to assess mizaj requires preliminary studies on conceptualization, weighting the indices (described in PM references), consensus building in history taking and physical examination, and using new approaches in personalized medicine.

## Figures and Tables

**Table 1:** Final included studies.

Authors	Date and Location of Study	Study Type	Population	Sample Size	Sample Age	Way of Mizaj Diagnosis	Type of Mizaj	Reliability and Validity of the Questionnaire	Number of Questions
Shahabi et al., 2007 * [8]	- Iran	Cross-sectional	Healthy person	37	20–40	Expert panel	Whole body mizaj	Not reported	-
Mojahedi et al., 2014 [5]	- Iran	Instrument design	Healthy person	52	20–40	Expert panel	Whole body mizaj	kappa coefficient: 0.4–0.82, Cronbach’s α coefficient: 0.71, content validity index of each item: 0.70–1.00	10
Sohrabvand et al., 2014 * [25]	2012 Iran	Cross-sectional	Infertile women	54	20–40	Self-designed questionnaire for uterine	Uterine and whole body mizaj	Cronbach’s alpha > 0.7	12
Mirtaheri et al., 2015 [26]	2013 Iran	Cross-sectional	Overweight women	135	18–30	Expert panel	Whole body mizaj	-	-
Parvizi et al., 2016 [21]	- Iran	Cross-sectional	Healthy person	86	20–40	Expert panel and Mojahedi’s Mizaj questionnaire	Whole body mizaj	kappa coefficient: 0.4–0.82, Cronbach’s α coefficient: 0.71, content validity index of each item: 0.70–1.00	10
Safari et al., 2016 * [27]	2014 Iran	Cross-sectional	Healthy person	109 healthy people	20–27	Mojahedi’s Mizaj questionnaire	Whole body mizaj	Cronbach’s alpha = 0.71	10
Dehnavi et al., 2016 *	2014–2015 Iran	Cross-sectional	People with premenstrual problems	65	20–40	Mojahedi’s Mizaj questionnaire	Whole body mizaj	Cronbach’s alpha = 0.71	10
Jafarnejad et al., 2016 [28]	2015 Iran	RCT	Women with premenstrual syndrome	Case = 35, control = 30	20–40	Mojahedi’s Mizaj questionnaire	Whole body mizaj	Cronbach’s alpha = 0.71	10
Roshandel et al., 2016 [29]	- Iran	Instrument design	Healthy person	197	18–70	Expert panel	Innate and acquired mizaj	Cronbach’s alpha = 0.912 for innate and 0.825 for acquired mizaj	First = 26, second = 56
Mohebbi et al., 2017 * [30]	2016 Iran	Cross-sectional	Healthy women	200	<20	Mojahedi’s Mizaj questionnaire	Whole body mizaj	Cronbach’s alpha = 0.71	10
Shakeri et al., 2017 * [31]	2014 Iran	Clinical trial	Healthy person	70	20–40	Mojahedi’s Mizaj questionnaire	Whole body mizaj	Cronbach’s alpha = 0.71	10
Zendehboodi et al., 2017 [32]	- Iran	-	Healthy male	247	20–40	Mojahedi’s Mizaj questionnaire	Whole body mizaj	kappa coefficient: 0.4–0.82, Cronbach’s α coefficient: 0.71, content validity index of each item: 0.70–1.00	10
Safari et al., 2017 * [33]	2014–2015 Iran	Cross-sectional	Healthy person	119	22.29 ± 2.02	Mojahedi’s Mizaj questionnaire	Whole body mizaj	Cronbach’s alpha = 0.71	10
Salmannezhad et al., 2017 [34]	2016 Iran	Cross-sectional	Healthy person	610	20–30	Mojahedi’s Mizaj questionnaire	Whole body mizaj	kappa coefficient: 0.4–0.82, Cronbach’s α coefficient: 0.71, content validity index of each item: 0.70–1.00	10
Zar et al., 2017 * [35]	2014 Iran	Cross-sectional	Healthy person	60	-	Mojahedi’s Mizaj questionnaire	Whole body mizaj	Cronbach’s alpha = 0.71	10
Mozaffarpur et al., 2017 [36]	- Iran	Cross-sectional	Healthy volunteers	150	18–40	Expert panel	Whole body mizaj	-	-
Tokaman nezhad et al., 2018 * [37]	2017 Iran	Cross-sectional	Pregnant women	169	Mean age = 27.7 ± 5.3	Mojahedi’s Mizaj questionnaire	Whole body mizaj	Cronbach’s alpha = 0.71	10
Salmannezhad et al., 2018 [20]	- Iran	Instrument design	Healthy person	221	20–60	Expert panel	Whole body mizaj	Cronbach’s alpha coefficient equal to 0.77–0.80	20
Hoseinzadeh et al., 2018 [23]	- Iran	Instrument design	Healthy person	10	-	Expert panel	Gastrointestinal dystemperament	Cronbach’s alpha = 0.795 validity equal to 0.8	49
Tavoosi et al., 2018 * [38]	2015–2017 Iran	Cross-sectional	Healthy person	293	22–24	Mojahedi’s Mizaj questionnaire	Whole body mizaj	Cronbach’s alpha = 0.71	10
Nematollahi et al., 2018 [39]	2016 Iran	Cross-sectional	Healthy volunteers	199	-	Mojahedi’s Mizaj questionnaire	Whole body mizaj	Cronbach’s alpha = 0.71	10
Parvizi et al., 2018 [40]	2016 Iran	Cross-sectional	Healthy person	112	20–40	Mojahedi’s Mizaj questionnaire	Whole body mizaj	Cronbach’s alpha = 0.71	10
Mojahedi et al., 2018 [41]	2016 Iran	Cross-sectional	Healthy person	74	19–40	Expert panel	Whole body mizaj	-	-
Bahman et al., 2018 [42]	2013–2015 Iran	Case study	Healthy women	150	18–45	Sohrabvand uterine questionnaire	Uterine temperament	Cronbach’s alpha > 0.7	12
Ilkhani et al., 2019 [43]	2015 Iran	Case–control	Type 1 diabetes mellitus patients and healthy controls	Case = 68, control = 80	Mean age = 10.0 ± 6.2	Mojahedi’s Mizaj questionnaire	Whole body mizaj	kappa coefficient: 0.4–0.82, Cronbach’s α coefficient: 0.71, content validity index of each item: 0.70–1.00	10
Moradi et al., 2019 [44]	2009–2010 Iran	Cross-sectional	PatientS with abnormal uterine bleeding	70	15–45	Questionnaire according to PM textbook	Uterine dystemperaments	Not reported	19
Banaei et al., 2019 * [45]	2017–2018 Iran	Cross-sectional	Healthy person	300	23 ± 4.48	Mojahedi’s Mizaj questionnaire	Whole body mizaj	Cronbach’s alpha = 0.71	10
Safari et al., 2019 * [46]	-	Cross-sectional	Healthy men	100	18<	Mojahedi’s Mizaj questionnaire	Whole body mizaj	Cronbach’s alpha = 0.71	10
Safari et al., 2019 * [47]	2013–2014 Iran	Cross-sectional	Healthy person	40	22.48 ± 5.4	Mojahedi’s Mizaj questionnaire	Whole body mizaj	Cronbach’s alpha = 0.71	10
Farhadinezhad et al., 2019 * [48]	- Iran	Cross-sectional	Healthy person	196	-	Salmannejad Mizaj questionnaire	Whole body mizaj	Cronbach’s alpha coefficient equal to 0.77–0.80	20
Rostami et al., 2019 * [49]	2016 Iran	Cross-sectional	113 prisoners, 113 non-prisonerS	226	20–40	Mojahedi’s Mizaj questionnaire	Whole body mizaj	Cronbach’s alpha = 0.71	10
Rajabzadeh et al., 2019 [50]	2017 Iran	Cross-sectional	Healthy men	105	18–35	Mojahedi’s Mizaj questionnaire	Whole body mizaj	Cronbach’s alpha = 0.71	10
Vahedi et al., 2020 * [51]	-	Cross-sectional	Diabetic patients	100 patients	18<	Mojahedi’s Mizaj questionnaire	Whole body mizaj	Cronbach’s alpha = 0.71	10
Tansaz et al., 2020 [22]	2013 Iran	Instrument design	Infertile females	54	20–40	Uterine mizaj questionnaire	Uterine mizaj	Cronbach’s alpha of 0.73 to 0.69	12
Farsani et al., 2020 [52]	- Iran	Cross-sectional	Healthy volunteers	45	18–40	Mojahedi’s Mizaj questionnaire	Whole body mizaj	Cronbach’s alpha = 0.71	10
Asghari et al., 2020 [53]	2016 Iran	Case–control	Healthy volunteers	30	20–40	Expert panel	Whole body mizaj	-	-
Kaviani et al., 2020 [54]	2018 Iran	Cross-sectional	Patients with abnormal uterine bleeding	112	20–40	Mojahedi’s Mizaj questionnaire	Whole body mizaj	Cronbach’s alpha = 0.71	10
Zareivash et al., 2020 * [55]	2019 Iran	Cross-sectional	Healthy person	165	20–60	Salmannejad Mizaj questionnaire	Whole body mizaj	Cronbach’s alpha coefficient equal to 0.77–0.80	20
Banaei et al., 2020 * [56]	2017–2018 Iran	Cross-sectional	Healthy person	296	23 ± 4.48	Mojahedi’s Mizaj questionnaire	Whole body mizaj	Cronbach’s alpha = 0.71	10
Mehr 2020 * [57]	2017 Iran	Cross-sectional	Healthy housewife	144	20–40	Mojahedi’s Mizaj questionnaire	Whole body mizaj	Cronbach’s alpha = 0.71	10
Aliabadi et al., 2021 [58]	2019 Iran	Cross-sectional	Healthy females	340	20–32	Salmannejad Mizaj questionnaire	Whole body mizaj	Cronbach’s alpha coefficient equal to 0.77–0.80	20
Aliabadi et al., 2021 [59]	- Iran	-	Healthy men	135	20–40	Mojahedi’s Mizaj questionnaire	Whole body mizaj	Cronbach’s alpha = 0.71	10
Mojahedi et al., 2021 * [60]	2015–2017 Iran	Instrument design	Diabetic children	-	-	Expert panel	Mizaj of diabetic child	-	11
Zendehboodi et al., 2021 [61]	2018 Iran	Case–control	Healthy person	Case = 110 Control = 181	>20	Mojahedi’s Mizaj questionnaire	Whole body mizaj	kappa coefficient: 0.4–0.82,Cronbach’s α coefficient: 0.71, content validity index of each item: 0.70–1.00	10
Khosrojerdi et al., 2021 * [62]	2017 Iran	Cross-sectional	60 healthy, 60 addictS)	120	25–32	Mojahedi’s Mizaj questionnaire	Whole body mizaj	Cronbach’s alpha = 0.71	10
Parvizi et al., 2022 [63]	- Iran	-	Healthy males	217	20–40	Mojahedi’s Mizaj questionnaire	Whole body mizaj	kappa coefficient: 0.4–0.82, Cronbach’s α coefficient: 0.71, content validity index of each item: 0.70–1.00	10
Noori et al., 2022 [64]	2020 Iran	Cross-sectional	Healthy person	145	26–60	Salmannejad Mizaj questionnaire	Whole body mizaj	Cronbach’s alpha coefficient equal to 0.77–0.80	20
Abbasian et al., 2022 [12]	2015–2017 Iran	Case–control	multiple sclerosis patients and healthy person	Case = 42, Control = 54	18–50	Expert panel and Mojahedi’s Mizaj questionnaire	Whole body and brain mizaj	kappa coefficient: 0.4–0.82, Cronbach’s α coefficient: 0.71, content validity index of each item: 0.70–1.00	10
Ghods et al., 2022 [65]	2020 Iran	Cross-sectional	Healthy person	34	Mean age = 37.11 ± 7	Mojahedi’s Mizaj questionnaire	Whole body mizaj	kappa coefficient: 0.4–0.82, Cronbach’s α coefficient: 0.71, content validity index of each item: 0.70–1.00	10
Nasiri et al., 2022 * [66]	2021 Iran	Descriptive study	COVID-19 patient	168 patientS	18–60	Salmannejad Mizaj questionnaire	Whole body mizaj	Cronbach’s alpha coeffcient equal to 0.77–0.80	20
Sultana et al., 2022 [24]	2019 India	Cross-sectional	People with amenorrhoea	80	14–50	Mojahedi’s Mizaj questionnaire	Whole body mizaj	Cronbach’s alpha = 0.71	10
Mozaffarpur et al., 2022 [67]	2020 Iran	Cross-sectional	Healthy volunteers	324	20–40	Expert panel	Whole body mizaj	-	-
Mojahedi et al., 2022 [11]	2016–2017 Iran	Cohort	Elderly person	1541	>60	Expert panel	Whole body mizaj	-	-
Razavi et al., 2022 [68]	2020 Iran	Cross-sectional	CTS patients	170	20<	Salmannejad Mizaj questionnaire	Whole body mizaj	Cronbach’s alpha coeffcient equal to 0.77–0.80	20

* These articles are in Persian.

**Table 2:** Details of articles with questionnaires.

Questionnaire	Type of Mizaj Assessment	Number of Items	Validity and Reliability	Number of Experts
Mojahedi et al., 2014 [5]	* WBM	10	kappa coefficient: 0.4–0.82, Cronbach’s α coefficient: 0.71, content validity index of each item: 0.70–1.00	10
Salmannezhad et al., 2018 [20]	* WBM	20	Cronbach’s alpha coefficient equal to 0.77–0.80	15
Hoseinzadeh et al., 2018 [23]	Dystemperament of gastrointestinal system	49	Cronbach’s alpha = 0.795 validity equalto 0.8	14
Tansaz et al., 2020 [22]	Uterine mizaj	12	Cronbach’s alpha of 0.73 to 0.69	1

* WBM = whole body mizaj.

## Data Availability

The data presented in the present systematic review are available upon request from the corresponding author.

## Supplementary Materials

The following supporting information can be downloaded at: https://www.mdpi.com/article/10.3390/diagnostics13050818/s1.
