# Supplementary material for: Assessment of the Diagnostic Methods of Mizaj in Persian Medicine: A Systematic Review
Source: Diagnostics (Basel). 2023 Feb 21;13(5):818. doi: 10.3390/diagnostics13050818 (PMC10000451; doi:10.3390/diagnostics13050818)
Supplement: Supplementary file 1 [file diagnostics-13-00818-s001.zip › diagnostics-2002786-supplementary.pdf]

Pubmed

((mizaj*[Title/Abstract] OR mezaj*[Title/Abstract] OR temperament*[Title/Abstract] OR dystemperament*[Title/Abstract] OR amzijah[Title/Abstract] OR akhlat[Title/Abstract] OR humor*[Title/Abstract] OR su-e-mizaj[Title/Abstract] OR su-e-mezaj[Title/Abstract] OR so-e-mezaj[Title/Abstract] OR so-e-mizaj[Title/Abstract])) AND ("Unanimedicine"[Title/Abstract] OR "Iranian medicine"[Title/Abstract] OR "Persian medicine"[Title/Abstract] OR "Iranian traditional medicine"[Title/Abstract] OR "traditional Iranian medicine"[Title/Abstract] OR "traditional medicine"[Title/Abstract] OR "unani concept"[Title/Abstract] OR "unani principle"[Title/Abstract] OR "complementary medicine"[Title/Abstract] OR "alternative medicine"[Title/Abstract])

Scopus

TITLE-ABS-KEY (mizaj* OR mezaj* OR temperament* OR dystemperament* OR amzijah OR akhlat OR humor* OR su-e-mizaj OR su-e-mezaj OR so-e-mezaj OR so-e-mizaj OR diagnos*) AND TITLE-ABS-KEY ("unani medicine" OR "iranian medicine" OR "persian medicine" OR "iranian traditional medicine" OR "traditional iranian medicine" OR "traditional medicine" OR "unani concept" OR "unani principle" OR "complementary medicine" OR "alternative medicine")

Web of science

(AB=(mizaj OR mezaj OR temperament OR dystemperament OR amzijah OR akhlat OR humor OR su-e-mizaj OR su-e-mezaj OR so-e-mezaj OR so-e-mizaj)) AND AB=("Unanimedicine" OR "Iranianmedicine" OR "Persianmedicine" OR "Iranian traditional medicine" OR "traditional Iranian medicine" OR "traditional medicine" OR "unani concept" OR "unani principle" OR "complementary medicine" OR "alternative medicine")

Scholar

(mizaj OR mezaj OR temperament OR dystemperament OR amzijah OR akhlat OR humor OR su-e-mizaj OR su-e-mezaj OR so-e-mezaj OR so-e-mizaj) AND ("Unani medicine" OR "Iranian medicine" OR "Persianmedicine" OR "Iranian traditional medicine" OR "traditional Iranian medicine" OR "traditional medicine" OR "unani concept" OR "unani principle" OR "complementary medicine" OR "alternative medicine")
